# Validity of the Single-Item Screen–Cannabis (SIS-C) for Cannabis Use Disorder Screening in Routine Care

**DOI:** 10.1001/jamanetworkopen.2022.39772

**Published:** 2022-11-01

**Authors:** Theresa E. Matson, Gwen T. Lapham, Jennifer F. Bobb, Malia Oliver, Kevin A. Hallgren, Emily C. Williams, Katharine A. Bradley

**Affiliations:** 1 Kaiser Permanente Washington Health Research Institute, Seattle; 2 Department of Health Systems and Population Health, University of Washington School of Public Health, Seattle; 3 Health Services Research & Development Center for Innovation for Veteran-Centered and Value-Driven Care, Veterans Affairs Puget Sound Health Care System, Seattle, Washington; 4 Department of Biostatistics, University of Washington School of Public Health, Seattle; 5 Department of Psychiatry and Behavioral Sciences, University of Washington School of Medicine, Seattle; 6 Department of Medicine, University of Washington School of Medicine, Seattle

## Abstract

**Importance:**

Cannabis use is prevalent and increasing, and frequent use intensifies the risk of cannabis use disorder (CUD). CUD is underrecognized in medical settings, but a validated single-item cannabis screen could increase recognition.

**Objective:**

To evaluate the Single-Item Screen–Cannabis (SIS-C), administered and documented in routine primary care, compared with a confidential reference standard measure of CUD.

**Design, Setting, and Participants:**

This diagnostic study included a sample of adult patients who completed routine cannabis screening between January 28 and September 12, 2019, and were randomly selected for a confidential survey about cannabis use. Random sampling was stratified by frequency of past-year use and race and ethnicity. The study was conducted at an integrated health system in Washington state, where adult cannabis use is legal. Data were analyzed from May 2021 to March 2022.

**Exposures:**

The SIS-C asks about frequency of past-year cannabis use with responses (none, less than monthly, monthly, weekly, daily or almost daily) documented in patients’ medical records.

**Main Outcomes and Measures:**

The *Diagnostic and Statistical Manual*, *Fifth Edition* (*DSM-5*) Composite International Diagnostic Interview–Substance Abuse Module (CIDI-SAM) for past-year CUD was completed on a confidential survey and considered the reference standard. The SIS-C was compared with 2 or more criteria on the CIDI-SAM, consistent with CUD. All analyses were weighted, accounting for survey design and nonresponse, to obtain estimates representative of the health system primary care population.

**Results:**

Of 5000 sampled adult patients, 1688 responded to the cannabis survey (34% response rate). Patients were predominantly middle-aged (weighted mean [SD] age, 50.7 [18.1]), female or women (weighted proportion [SE], 55.9% [4.1]), non-Hispanic (weighted proportion [SE], 96.7% [1.0]), and White (weighted proportion [SE], 74.2% [3.7]). Approximately 6.6% of patients met criteria for past-year CUD. The SIS-C had an area under receiver operating characteristic curve of 0.89 (95% CI, 0.78-0.96) for identifying CUD. A threshold of less than monthly cannabis use balanced sensitivity (0.88) and specificity (0.83) for detecting CUD. In populations with a 6% prevalence of CUD, predictive values of a positive screen ranged from 17% to 34%, while predictive values of a negative screen ranged from 97% to 100%.

**Conclusions and Relevance:**

In this diagnostic study, the SIS-C had excellent performance characteristics in routine care as a screen for CUD. While high negative predictive values suggest that the SIS-C accurately identifies patients without CUD, low positive predictive values indicate a need for further diagnostic assessment following positive results when screening for CUD in primary care.

## Key Points

**Question:**

Is a single question about cannabis use, used in routine primary care with results documented in electronic health records, an accurate screening test for past-year cannabis use disorder?

**Findings:**

In this diagnostic study of 1688 adult patients, the Single-Item Screen–Cannabis (SIS-C) had excellent performance characteristics as a screening test for cannabis use disorder. Report of any past-year cannabis use balanced sensitivity and specificity.

**Meaning:**

These findings suggest that the brief SIS-C is a valid screen for cannabis use disorder and could be easily integrated with screening for other behavioral health conditions in primary care.

## Teaser

This diagnostic study evaluates the Single-Item Screen–Cannabis (SIS-C), a single-question measure to assess frequency of cannabis use in routine primary care, to detect past-year cannabis use disorder (CUD).

## Introduction

Nearly 50 million people in the United States use cannabis,^1^ reflecting a trend toward increasing use and decreasing perception of risk.^2^ Among primary care patients in Washington state, where adult cannabis use is legal, more than 20% report past-year cannabis use.^3,4^

Frequent cannabis use increases the risk of a cannabis use disorder (CUD),^5^ a pattern of continued cannabis use despite clinically significant impairment and distress.^6,7^ The prevalence of CUD ranges from 2% to 5% in the general population,^8,9,10,11^ 5% to 14% in young adults,^9,11^ and 8% to 23% among those with mental health or other substance use disorders.^10,12^ CUD is the largest contributor to cannabis-attributable disease burden,^13^ including injuries related to cannabis intoxication (eg, motor vehicle accidents),^14^ worsened mental health symptoms (eg, psychosis),^15^ other substance use disorders,^10^ other medical conditions (eg, bronchial system problems),^16^ and adverse pregnancy outcomes.^7,13,17^ Despite evidence-based treatment (ie, behaviorally based therapies),^18,19,20,21^ CUD remains underrecognized and largely untreated.^1,10,22^

A brief, valid cannabis screen could increase identification of CUD, but it must be feasible for general medical settings with limited visit time. To our knowledge, no study has tested the validity of cannabis screens administered in routine care and documented in the electronic health record (EHR). Optimal screening tools for general medical settings typically include fewer than 4 items,^23,24^ with single items recommended.^25,26^ Single-item screens can be integrated with other behavioral health screens to increase the feasibility of routine CUD screening.^27^ Although validated single-item screens can identify substance use disorders generally, none are specific to CUD.^28,29,30,31^ Increasing cannabis use and legalization underscore the need to screen for cannabis separately from other substances.^32^ One health system integrated a question about the frequency of past-year cannabis use into routine care at the request of frontline clinicians.^32^ This study evaluated the performance of that Single-Item Screen–Cannabis (SIS-C) when documented in the EHR as part of routine care.

## Methods

### Setting

This prospective diagnostic study follows Standards for Reporting of Diagnostic Accuracy (STARD) reporting guideline and took place at Kaiser Permanente Washington (KPWA), an integrated health care system providing health insurance and medical care in Washington state, where adult cannabis use is legal. KPWA conducts annual population-based screening for behavioral health conditions (depression,^33^ alcohol,^34^ cannabis, and other drug use) in primary care using a 7-item questionnaire, with results documented in the EHR.^27^ A single item, the SIS-C (described in the Measures subsection), asks patients about the frequency of past-year cannabis use. Responses to the SIS-C trigger additional assessment for CUD, guiding clinical decision-making.

### Sample

Adult patients (aged ≥18 years) at KPWA who completed the SIS-C in primary care between January 28 and September 12, 2019 (N = 108 950) were eligible to be sampled for a confidential cannabis survey (eFigure 1 in the Supplement). Patients were ineligible if they were current or recent KPWA employees (approximately 4%), needed an interpreter (2.6%), lived outside Washington state (<1%), were deceased (<1%), or opted out of research (<1%). Using EHR data, 5000 patients were randomly sampled for the survey. As detailed elsewhere,^35^ we oversampled for higher frequency of past-year cannabis use (58% daily, 24% weekly, 7% monthly, 6% less than monthly, 6% no use) and ensured 35% of the sample were individuals who belonged to minoritized racial and ethnic groups to obtain representation from important subgroups.

### Procedures

Patients were recruited within 60 days of the SIS-C to ensure proximity of screen and survey responses. Patients were mailed invitations with information about the study, confidentiality, and unique identifiers linking responses to participants’ EHRs. Reminders were offered by telephone and email. The survey took approximately 20 minutes to complete online (63%) or by telephone (34%). Participants acknowledged informed consent online or by telephone prior to the survey and received $20 compensation.

The study sample included 1688 primary care patients who completed the survey, representing a 34% response rate, consistent with current health survey research.^36,37^ The KPWA Health Research Institute Institutional Review Board approved this study with waivers of consent (to identify eligible sample), consent documentation (for survey respondents), and HIPAA authorization.

### Measures

#### Reference Standard for Past-Year CUD

The Composite International Diagnostic Interview Substance Abuse Module (CIDI-SAM) was selected as the reference standard for *Diagnostic and Statistical Manual of Mental Disorders*, *Fifth Edition* (*DSM-5*) CUD based on demonstrated feasibility of administration.^38,39^ The 15-item CIDI-SAM provides a diagnosis and scaled score of CUD severity (0-11), reflecting the number of *DSM-5* CUD criteria met. Any past-year CUD (mild to severe) was defined as 2 or more CUD criteria endorsed on the CIDI-SAM; moderate to severe past-year CUD was defined as 4 or more CUD criteria, consistent with the *DSM-5*.^6^ The first 2 survey questions asked about frequency and recency of cannabis use. Patients who reported no past-year use on both did not complete the CIDI-SAM to minimize assessment burden (n = 94). These patients were assigned a CIDI-SAM score of zero.

#### EHR-Documented SIS-C

The SIS-C, offered as part of routine primary care, asked about frequency of past-year cannabis use (“How often in the past year did you use marijuana?”) with response options (“never,” “less than monthly,” “monthly,” “weekly,” and “daily or almost daily”), adapted from the third question of the World Health Organization’s Alcohol Use Disorders Identification Test,^40^ and scored from 0 to 4 points. The term *marijuana* was not defined and could include medical and nonmedical use—relevant in cannabis-legal settings where medical authorization by a clinician is not required. The SIS-C was embedded in the 7-item behavioral health questionnaire,^27,41^ self-administered on paper during the study period. An electronic flag prompted administration of the screen after check-in if patients had not been screened in the past year, and a medical assistant entered responses into the EHR before the physician visit.

#### Sociodemographic Characteristics and Comorbidities

Demographic information collected from patients and documented in the EHR by the health system at the time of sampling was used to approximate the social conditions that shape the health of patients at different developmental stages^42^ and with different lived experiences (sexism,^43,44,45^ racism,^46,47,48^ other social determinants of health^49^). This included age (18-29, 30-49, 50-64, or ≥65 years), sex or gender (female or woman or male or man; this administrative field may reflect biological sex or gender identity), race (American Indian or Alaska Native, Asian, Black or African American, Native Hawaiian or Pacific Islander, White, other, or unknown), and ethnicity (Hispanic or non-Hispanic). Due to small sample sizes and/or low prevalence of CUD in certain subgroups, we combined age groups (18-29, 30-49, ≥50 years)^50^ and race and ethnicity (non-Hispanic Black, non-Hispanic White, Hispanic)^51^ for subgroup analyses. Socioeconomic status was approximated using insurance status from enrollment records, marital status, education, employment, and type of residence reported on survey. Mental health and substance use disorder diagnoses were based on *International Classification of Disease, Tenth Revision* codes documented in the EHR or insurance claims in the year prior to survey completion.

### Statistical Analysis

#### Survey Weighting

All analyses were weighted to account for stratified random sampling and nonresponse. Specifically, we calculated sampling weights by taking the inverse probability of being sampled within 10 sampling strata resulting from the 5 cannabis screen responses and indicator for patients who belong to minoritized racial and ethnic groups.^52,53^ We calculated nonresponse weights using logistic regression to estimate inverse probabilities of survey nonresponse based on sociodemographic characteristics.^54^ We multiplied sampling weights and nonresponse weights to obtain estimates representative of the KPWA primary care population.^55^ Comparisons of the eligible primary care population, eligible survey sample, nonrespondents, respondents, and the weighted primary care sample have been previously reported.^35^

#### Descriptive and Screening Test Performance Characteristics

We described characteristics of the sample, including the frequency of each response option on the SIS-C. We compared the SIS-C with the reference standard of any CUD and moderate to severe CUD. To assess screening test performance characteristics, we estimated sensitivity (true-positive rate) and specificity (true-negative rate) for each cut point on the SIS-C.^56,57^ We depicted receiver operating characteristic (ROC) curves graphically and estimated area under the curves (AUCs). ROC curves provide a useful summary of the overall discriminatory power of a screening test.^56^ AUCs can range from 0 to 1.0, with 0.8 to 0.9 considered excellent performance and greater than 0.9 considered outstanding.^58,59^ We estimated 95% CIs for weighted AUC estimates using nonparametric bootstrapping with 10 000 replications.^60^

#### Differences Across Sociodemographic Subgroups

To determine whether the SIS-C performs differently across sociodemographic subgroups, we plotted ROC curves stratified by age, sex or gender, and race and ethnicity. We evaluated differences between AUCs across subgroups and bootstrapped 95% CIs for differences; 95% CIs that did not contain zero indicated statistically significant between-group differences.^60^

#### Predictive Value of SIS-C for CUD

Although positive and negative predictive values (indicating the probability of a condition given a positive screening result and absence of a condition with a negative screening result) are often reported in validation studies, these postscreening probabilities are highly dependent on the prevalence of the condition in the screened population.^61^ Using Bayes theorem,^61^ we modeled the probability a patient has CUD if the SIS-C is positive and the probability a patient does not have CUD if the SIS-C is negative across a range of prevalence estimates for CUD (<0.5%-30%)^8,9,10,11,12^ to understand performance when applied to different populations. Analyses used Stata version 15.1 (StataCorp),^62^ R version 4.0.2 (R Project for Statistical Computing), and Excel version 2202 (Microsoft Corp) and were conducted from May 2021 to March 2022.

## Results

Table 1 describes the sample, which was weighted to reflect the eligible primary care population screened for cannabis use. The weighted sample was predominantly middle-aged (weighted mean [SD] age, 50.7 [18.1]), female or women (weighted proportion [SE], 55.9% [4.1]), non-Hispanic (weighted proportion [SE], 96.7% [1.0]), White (weighted proportion [SE], 74.2% [3.7]), married or living with a partner (weighted proportion [SE], 62.8% [4.0]), with indicators of higher socioeconomic status. More than 25% had mental health diagnoses, and 5% had substance use disorder diagnoses. Based on the survey-administered CIDI-SAM reference standard, 6.6% of primary care patients met criteria for any past-year *DSM-5* CUD and 1.9% for moderate to severe CUD. Characteristics stratified by CUD are available in eTable 1 in the Supplement.

**Table 1.** Characteristics of the Eligible Primary Care Population

Patient characteristics	Unweighted, No. (N = 1688)	Weighted % (SE)
Age^a^		
18-29	459	14.9 (2.8)
30-49	582	31.0 (3.9)
50-64	329	26.3 (3.8)
≥65	318	27.7 (3.4)
Sex or gender^a^		
Female or women	861	55.9 (4.1)
Male or men	827	44.1 (4.1)
Race^a^		
American Indian or Alaska Native	40	0.4 (0.0)
Asian	99	9.7 (2.4)
Black or African American	163	4.8 (1.7)
Native Hawaiian or Pacific Islander	36	2.4 (0.1)
White	1192	74.2 (3.7)
Other or unknown race^b^	158	8.4 (2.5)
Hispanic ethnicity^a^	174	3.3 (1.0)
Insurance^a^		
Medicaid or subsidized	210	6.0 (1.8)
Medicare	323	27.1 (3.4)
Commercial	1072	64.9 (3.7)
Unknown	83	2.0 (0.8)
Marital status^c^		
Married or living with partner	966	62.8 (4.0)
Widowed	43	3.0 (1.3)
Divorced or separated	166	9.2 (2.4)
Single or never married	505	24.1 (3.5)
Missing	8	0.9 (0.8)
Education^c^		
≤High school	319	12.7 (2.7)
Some college	665	38.6 (4.0)
≥4 Years of college	694	47.8 (4.1)
Missing	10	0.9 (0.8)
Employment^c^		
Employed full time	988	55.4 (4.1)
Employed part time	152	12.6 (2.9)
Retired	298	22.0 (3.1)
Other	186	8.3 (2.3)
Unemployed	58	0.8 (0.2)
Missing	6	0.9 (0.8)
EHR-documented past-year diagnoses^a^		
Mental health diagnosis	612	26.5 (3.6)
SUD diagnosis	106	5.2 (2.0)
Mental health or SUD diagnosis	662	28.7 (3.7)
CIDI-SAM criteria for cannabis use disorder^c^^,^^d^		
<2, No CUD	1070	93.3 (1.0)
2-3, Mild CUD	364	4.7 (0.9)
≥4, Moderate to severe CUD	254	1.9 (0.2)

Abbreviations: CIDI-SAM, Composite International Diagnostic Interview Substance Abuse Module; CUD, cannabis use disorder; EHR, electronic health record; SUD, substance use disorder.

^a^ Data collected from EHRs.

^b^ Patients are provided the option to indicate other when choosing among 1 or more race categories at appointing or check-in.

^c^ Data collected from confidential survey.

^d^ Participants who reported no past-year cannabis use on the survey were assigned a score of 0 on the CIDI-SAM.

### Identification of Any Past-Year CUD

The SIS-C had excellent performance characteristics as a screen for any past-year CUD (Table 2), with an AUC of 0.89 (95% CI, 0.78-0.96) (Figure). Report of any cannabis use (ie, less than monthly or more frequent use) on the SIS-C balanced sensitivity (88%) and specificity (83%).

**Table 2.** Prevalence and Performance Characteristics of the Single-Item Screen–Cannabis for Identification of Past-Year Cannabis Use Disorder

Potential cut points for the Single-Item Screen-Cannabis^c^	Prevalence of response		Screening performance for past-year CUD					
	Unweighted, No.	Weighted, % (SE)	Any CUD^a^			Moderate-Severe CUD^b^		
			Sensitivity, %	Specificity, %	AUC (95% CI)^d^	Sensitivity, %	Specificity, %	AUC (95% CI)^d^
Never	99	78.1 (2.0)	NA	NA	0.89 (0.78-0.96)	NA	NA	0.95 (0.94-0.96)
≥Less than monthly	99	9.6 (1.2)	88	83		100	80	
≥Monthly	118	3.3 (0.4)	71	92		96	89	
≥Weekly	376	4.0 (0.4)	57	94		81	92	
Daily or almost daily	996	5.1 (0.4)	36	97		57	96	

Abbreviations: AUC, area under the receiver operating characteristic curve; CUD, cannabis use disorder; NA, not applicable.

^a^ Endorsed 2 or more criteria on the Composite International Diagnostic Interview–Substance Abuse Module.

^b^ Endorsed 4 or more criteria on the Composite International Diagnostic Interview–Substance Abuse Module.

^c^ The Single-Item Screen–Cannabis asked, “How often in the past year did you use marijuana?” with responses documented in the electronic health record as part of routine care.

^d^ The 95% CIs were obtained using nonparametric bootstrapping of weighted AUC estimates.

**Figure.**
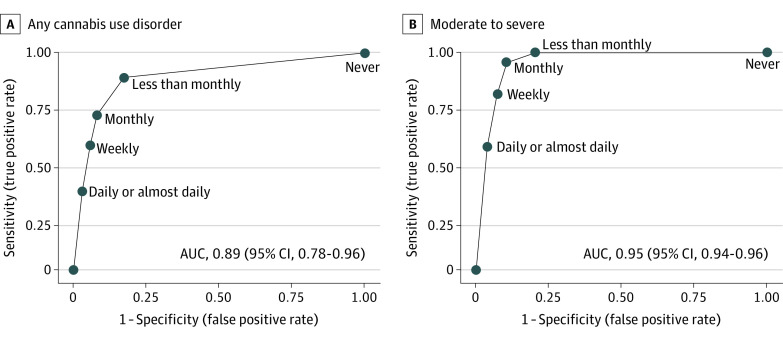
Receiver Operating Characteristic Curves for the Single-Item Screen–Cannabis Compared With the Reference Standard for Past-Year Cannabis Use Disorder The Single-Item Screen–Cannabis is administered annually to primary care patients and asks about the frequency of past-year cannabis use (never, less than monthly, monthly, weekly, daily or almost daily). The Composite International Diagnostic Interview–Substance Abuse Module was used as the reference standard for past-year *Diagnostic and Statistical Manual*, *Fifth Edition* cannabis use disorder and was administered to survey participants on a confidential survey. The Single-Item Screen–Cannabis was compared with any cannabis use disorder (Composite International Diagnostic Interview–Substance Abuse Module ≥2) and moderate-severe cannabis use disorder (Composite International Diagnostic Interview–Substance Abuse Module ≥4).

The probability of any past-year CUD based on a positive SIS-C (positive predictive value) varied across the range of population-based prevalences and screening thresholds (Table 3). If the underlying prevalence of CUD were 4% in the screened population, the probability of CUD in patients with positive SIS-C screens ranged from 12% to 26% across screening thresholds (less than monthly to daily or almost daily). The probability of no CUD in patients with negative SIS-C screens (negative predictive value) ranged from 98% to 100%. If the population prevalence of CUD were 8% (eg, prevalence among young men), the probability of CUD in patients with positive SIS-C screens ranged from 22% to 42%, and the probability of no CUD for patients with negative SIS-C screens ranged 95% to 100%. If the prevalence of CUD were 20% to 30% (eg, prevalence among patients with mental health or substance use disorders), the probability of CUD in patients with positive SIS-C screens ranged from 45% to 78%, and the probability of no CUD in patients with negative SIS-C screens ranged from 81% to 100%.

**Table 3.** Probability of Past-Year CUD if SIS-C Is Positive (or Negative) Across a Range of Population-Based Prevalence Estimates for CUD

Threshold for positive SIS-C	Population prevalence^a^							
	0.5%	2.0%	4.0%	6.0%	8.0%	10.0%	20.0%	30.0%
**Any CUD**								
Probability patient has CUD if SIS-C is positive, %								
≥Less than monthly	1.6	6.3	12.1	17.4	22.4	26.9	45.3	58.7
≥Monthly	2.6	9.9	18.4	25.7	32.0	37.5	57.5	69.9
≥Weekly	2.8	10.3	19.0	26.5	32.9	38.5	58.5	70.7
Daily or almost daily	4.0	14.4	25.5	34.4	41.7	47.7	67.3	77.9
Probability patient does not have CUD if SIS-C is negative, %								
≥Less than monthly	100.0	100.0	100.0	100.0	100.0	100.0	99.9	99.9
≥Monthly	99.9	99.7	99.4	99.1	98.8	98.4	96.6	94.2
≥Weekly	99.8	99.2	98.4	97.6	96.8	95.9	91.3	85.9
Daily or almost daily	99.7	98.9	97.8	96.7	95.5	94.4	88.1	81.2
**Moderate to severe CUD**								
Probability patient has CUD if SIS-C is positive, %								
≥Less than monthly	1.5	5.7	11.1	16.0	20.6	24.9	42.7	56.1
≥Monthly	2.4	9.1	16.9	23.7	29.8	35.2	55.0	67.7
≥Weekly	3.0	11.1	20.4	28.2	34.8	40.6	60.6	72.5
Daily or almost daily	4.5	16.1	28.2	37.5	45.0	51.1	70.2	80.1
Probability patient does not have CUD if SIS-C is negative, %								
≥Less than monthly	100.0	100.0	100.0	100.0	100.0	100.0	100.0	100.0
≥Monthly	100.0	99.9	99.8	99.7	99.5	99.4	98.7	96.5
≥Weekly	99.9	99.7	99.3	99.0	98.6	98.2	96.1	90.2
Daily or almost daily	99.8	99.4	98.8	98.1	97.4	96.7	93.0	83.2

Abbreviations: CUD, cannabis use disorder; SIS-C, Single-Item Screen–Cannabis.

^a^ Range of prevalence estimates for past-year *Diagnostic and Statistical Manual for Mental Disorders, Fifth Edition,* CUD was based on prior literature finding the overall prevalence of any CUD to be 2% to 4% and the overall prevalence of moderate to severe CUD to be 1% to 2%, with higher prevalence estimates for some subgroups (eg, men, young adults, patients with mental health and substance use disorder) and lower prevalence estimates for some subgroups (eg, women, older adults).

### Identification of Moderate to Severe CUD

The SIS-C had outstanding performance characteristics for past-year moderate to severe CUD, with an AUC of 0.95 (95% CI, 0.94-0.96) (Figure). Report of monthly or more frequent cannabis use balanced sensitivity (96%) and specificity (89%). Report of daily or almost daily cannabis use had high specificity (96%) but lower sensitivity (57%) (Table 2).

The probability of past-year moderate to severe CUD based on a positive or negative SIS-C screen varied across the range of population-based prevalences and screening thresholds (Table 3). Probabilities were slightly higher than those reported for any CUD.

### Performance Across Sociodemographic Subgroups

There were statistically significant but small differences in the performance of the SIS-C across age and race and ethnicity. Full results appear in the eAppendix, eFigure 2, and eTable 2 in the Supplement.

## Discussion

This study evaluated the screening performance of the SIS-C, a single-item cannabis screen administered and documented in the EHR as part of routine primary care in a US state where adult cannabis use is legal. Among primary care patients, 6.6% met criteria for past-year *DSM-5* CUD based on the confidential CIDI-SAM reference standard, slightly higher than national survey estimates.^8,9,10,11^ The SIS-C had excellent performance characteristics as a screening test for any past-year CUD and outstanding performance characteristics for moderate to severe CUD. Report of any past-year cannabis use balanced sensitivity and specificity for any CUD, whereas report of monthly or more frequent cannabis use balanced sensitivity and specificity for moderate to severe CUD.

While there are several substance use screens validated in general adult patient populations,^23,28,29,30,31,39,63,64,65,66,67^ few are single-item,^28,29,30,31^ no single-item screens are specific to cannabis use, and none have been validated when administered during routine care and documented in the medical record (Table 4). Existing single-item screens^28,29,30,31^ combine cannabis screening with other illegal drugs. In the context of legalization and increasing prevalence, clinicians may want to screen for cannabis separately from other drugs, as is recommended for alcohol.^27^ One previously validated brief screen includes cannabis-specific items, but only in the second stage of a 2-stage screening process.^39^ Other previously validated cannabis-specific screens (Cannabis Abuse Problems Identification Test [CUPIT], Cannabis Use Disorder Identification Test [CUDIT], CUDIT-Revised) provide more detail but may not be practical for use in routine care due to their length (>4 items)^68,69,70^ or appropriate due to validation only in people who use cannabis (CUDIT–Short Form, Severity of Dependence Scale).^71,72^

**Table 4.** Review of Brief Validated Substance Use Screens (≤4 Items) for Current Cannabis or Other Drug Use Disorder in Adults in a General Medical Setting

Screen^a^	Items, No.	2-Step screen	Cannabis-specific items, No.	Validated for CUD	Validated for SUD	Administration	Research or routine care	Optimal cut-point (range)	Sensitivity, %	Specificity, %	AUC
SIS-C	1	No	1	Yes (CIDI-SAM)	No	Self	Routine care	Any CUD: ≥less than monthly; moderate to severe CUD: ≥monthly (never to daily)	Any CUD: 88; moderate to severe CUD: 96	Any CUD: 83; moderate to severe CUD: 89	Any CUD: 0.89; moderate to severe CUD: 0.95
SoDU^23,63^	1-2 (+1 to ascertain cannabis use)	Yes	0	Yes (MINI)	Yes (MINI)	Interview	Research	SUD and CUD: item 1, ≥7 or item 2, ≥2 (0-365 d)	SUD: 92; CUD: 100	SUD: 93; CUD: 88	SUD: 0.93; CUD: 0.94
TAPS^39^	4-30	Yes	3	No	Yes (CIDI-SAM)	Interview	Research	SUD in cannabis use sample: positive TAPS-1 and TAPS-2 ≥2 (0-3)	71	95	NA
TAPS-1^28^	4 (1 item for illegal drugs)	No	0	No	Yes (WMH-CIDI)	Self and interview	Research	Any SUD and moderate to severe SUD: ≥less than monthly (never to daily)	Any SUD: 93; moderate to severe SUD: 95	Any SUD: 85; moderate to severe SUD: 83	Any SUD: 0.89; moderate to severe SUD: 0.89
ASSIST-Drug^65^	1-2	Yes	0	No	Yes (MINI)	Interview	Research	DUD: item 1, ≥2 (0-365 d) or item 2, ≥5 (0-365 d)	95	88	0.92
DAST-2^64^	2	No	0	No	Yes (MINI)	Interview	Research	DUD: ≥1 (0-365 d)	94	89	0.92
RDPS^67^	4 (+1 to ascertain cannabis use)	Yes	0	No	Yes (CIDI-SAM and *ICD-10*)	Interview	Research	Drug abuse or dependence: ≥1 (0-4); drug dependence: same as previous	Drug abuse or dependence: 81; drug dependence: 83	Drug abuse or dependence: 98; drug dependence: 97	Drug abuse or dependence: 0.90; drug dependence: 0.91
SSIQ^29^	1	No	0	No	Yes (MINI-Plus)	Self	Research	SUD: ≥1 (0 to ∞ times)	85	89	0.87
SQST^31^	1	No	0	No	Yes (CIDI-SAM)	Interview	Research	DUD: ≥1 (0 to ∞ times)	100	74	NA
SUBS^30^	4 (1 item for illegal drugs)	No	0	No	Yes (MINI-Plus)	Self	Research	SUD: ≥1 or 2 d (never to 3 or more days)	82	89	0.85
TICS^66^	2	No	0	No	Yes (CIDI-SAM)	Interview	Research	SUD: any reported use (never to often)	79	78	NA

Abbreviations: ASSIST-Drug, Alcohol, Smoking and Substance Involvement screening Test–Drug; AUC, area under receiver operating characteristic curves; CIDI-SAM, Composite International Diagnostic Interview–Substance Abuse Module; CUD, cannabis use disorder; DAST-2, Drug Abuse Screening Test; DUD, drug use disorder; *ICD-10*, *International Statistical Classification of Diseases and Related Health Problems, Tenth Revision*; MINI, Mini-International Neuropsychiatric Interview; NA, not applicable; RDPS, Rapid Drug Problem Screen; SIS-C, Single-Item Screen–Cannabis; SoDU, Screen of Drug Use; SQST, Single-Question Screening Test; SSIQ, Single-Item Screening Questions; SUBS, Substance Use Brief Screen; SUD, substance use disorder; TAPS, Tobacco, Alcohol, Prescription Medication, and Other Substance Use; TICS, Two-Item Conjoint Screen; WMH-CIDI, World Mental Health–Composite International Diagnostic Interview.

^a^ Screens were excluded if they were not validated (eg, National Institutes of Drug Abuse Quick Screen, Drug History Questionnaire or Psychoactive Drug History Questionnaire), had more than 4 items (eg, Cannabis Use Disorder Identification Test–Revised, Cannabis Abuse Problems Identification Test, Cannabis Abuse Screening Test), did not assess current use (eg, Cut Down, Annoyed, Guilty, Eye-Opener–Adapted to Include Drugs), validated only among people who use cannabis (eg, Cannabis Use Disorder Identification Test–Short Form, Severity of Dependence Scale), adolescents (eg, Screen to Brief Intervention, Brief Screener for Tobacco, Alcohol and Other Drugs), or in a specialty population such as pregnant women (eg, Substance Use Risk Profile-Pregnancy).

This is the first study, to our knowledge, to evaluate the performance characteristics of any substance use screen when used in routine clinical care. Patients may respond differently to substance use screens when administered in clinical settings—where clinicians will see results in the medical record—compared with when administered in confidential research settings. It is promising, therefore, that the performance of the EHR-documented SIS-C for any CUD was comparable with the performance of single-item drug screens validated in research settings,^28,29,30,31^ and its performance for identifying moderate to severe CUD was stronger.^28^ Approximately 90% of KPWA primary care patients are screened annually with the SIS-C, demonstrating routine use is feasible and clinically useful.

The SIS-C performed well across all groups based on age, sex or gender, and race and ethnicity, but performance characteristics were less strong for younger and middle-aged adults relative to older adults. Because young adults have a higher prevalence of CUD and may be more susceptible to risks of CUD,^73^ the lowest threshold on the SIS-C (any use) may be preferred. Performance characteristics were also less strong for Hispanic patients relative to non-Hispanic White patients. Hispanic patients may underreport cannabis use to avoid repercussions^74^ stemming from intersecting cannabis and anti-immigration stigma.^48^

Selection of a SIS-C screening threshold for detecting CUD will depend on the prevalence of CUD in the setting where screening is taking place and resources for follow-up assessment and care. Although any use and monthly use were the optimal screening thresholds for identifying any CUD and moderate to severe CUD, respectively, applying Bayes theorem, we found that the probability a patient with a positive screen has CUD was low when the underlying prevalence of CUD in the screened population was less than 8%. A lower threshold, such as any use, may be appropriate for some settings (eg, mental health) and populations (eg, young men) expected to have a higher prevalence of CUD^9,10,11,12^; whereas a higher threshold, such as daily use, may be appropriate for general medical settings.^8,9,10^ Threshold selection also depends on how a positive screen will be used, the costs of false-positive results, and the benefits of true-positive results.^75,76^ Costs of screening include time and resources to administer further assessment and inappropriate diagnostic labeling of patients.^34^ Benefits of screening include identifying at-risk patients for prevention and harm reduction, clinician recognition of underlying reasons for cannabis use (eg, chronic pain, insomnia, depression, anxiety)^35,77^ and treatment options.^19^ Prioritizing a sensitive threshold may be appropriate as part of behavioral health screening in primary care settings when the screen is followed by low-cost, low-burden, nonstigmatizing symptom assessment and discussion of symptoms.^27,41^ Prioritizing a specific threshold that minimizes false positive screens might be more appropriate in resource-constrained settings or those in which a positive screen results in referral.^57^

The SIS-C is not a replacement for assessment of CUD symptoms or for making a diagnosis. Many—or most—patients who screen positive on the SIS-C will not meet criteria for CUD, as reflected by low positive predictive values. This is common when the underlying prevalence of the screened condition is low. Follow-up administration of longer assessments using *DSM-5* criteria will be important for diagnosis of CUD.^78^ Furthermore, the SIS-C provides a starting point for asking patients about cannabis use to support clinicians in exploring reasons for use—including medical reasons^79^—and discussing benefits and risks of use.^80^ Finally, high negative predictive values suggest that the SIS-C accurately identifies patients without CUD so that patients who screen negative need no further evaluation.

### Limitations

This study has limitations. While the CIDI-SAM is considered a criterion-standard measure of CUD,^38,39^ in-person administration and urine drug screening were not feasible. We used the entire survey sample, assuming no CUD criteria for respondents who indicated no past-year use on 2 different questions, to increase representation across the spectrum of cannabis use and minimize spectrum bias.^81^ This approach could introduce measurement error, but we expected minimal bias, as participants indicated no past-year cannabis use twice. The CIDI-SAM asked about “cannabis” use, whereas the SIS-C asked about “marijuana”; it is unclear whether patients interpreted these terms synonymously or whether they considered medical use and/or other cannabis products in their response on the SIS-C. Only 34% of invited patients completed the survey. Although lower than desired, this response rate is consistent with industry averages^35^ and reflects a national trend of declining response rates.^82^ Consequently, responses from a small number of patients may have contributed disproportionately to analyses due to weighting for oversampling design and nonresponse.^83^ These weights were estimated using measured factors and cannot account for unmeasured factors; however, we found patient characteristics of the weighted sample were similar to the eligible primary care population^35^ and other KPWA patients overall.^3^ We were unable to conduct subgroup analyses for all age and race subgroups due to small sample sizes. Additionally, this study was conducted in a state with legal cannabis use and among patients who were largely White, commercially insured, and with high socioeconomic status, potentially limiting generalizability. Future studies are needed to evaluate the SIS-C in settings where adults may experience legal consequences for cannabis use.

## Conclusions

This study found that the SIS-C—a self-administered, single-item screen for CUD—had excellent performance characteristics when used in routine primary care in a setting with legal cannabis use. The screening thresholds can be tailored to patient populations and the needs and preferences of health settings. The SIS-C can be easily integrated with other behavioral health screening,^27,41^ making screening for CUD feasible in primary care.

## Notes

Supplement. **eFigure 1.** Flow Diagram of Study Sample **eTable 1.** Characteristics of the Eligible Primary Care Population (N=1688), Stratified by Severity of Past-Year Cannabis Use Disorder (CUD) **eAppendix. **Performance of the SIS-C Across Sociodemographic Subgroups **eFigure 2.** Receiver Operating Characteristic (ROC) Curves for the Single-Item Screen–Cannabis (SIS-C) Compared With the Reference Standard for Past-Year Cannabis Use Disorder (CUD), Stratified by Subgroups **eTable 2.** Differences in Area Under the Receiver Operating Characteristic Curve (AUC) Estimates Comparing Performance of the SIS-C Between Demographic Subgroups and Performance SIS-C With Other Survey Measures
