# Supplementary material for: Validity of the Single-Item Screen–Cannabis (SIS-C) for Cannabis Use Disorder Screening in Routine Care
Source: JAMA Netw Open. 2022 Nov 1;5(11):e2239772. doi: 10.1001/jamanetworkopen.2022.39772 (PMC9627408; doi:10.1001/jamanetworkopen.2022.39772)
Supplement: Supplement. — eFigure 1. Flow Diagram of Study Sample eTable 1. Characteristics of the Eligible Primary Care Population (N=1688), Stratified by Severity of Past-Year Cannabis Use Disorder (CUD) eAppendix. Performance of the SIS-C Across Sociodemographic Subgroups eFigure 2. Receiver Operating Characteristic (ROC) Curves for the Single-Item Screen–Cannabis (SIS-C) Compared With the Reference Standard for Past-Year Cannabis Use Disorder (CUD), Stratified by Subgroups eTable 2. Differences in Area Under the Receiver Operating Characteristic Curve (AUC) Estimates Comparing Performance of the SIS-C Between Demographic Subgroups and Performance SIS-C With Other Survey Measures [file jamanetwopen-e2239772-s001.pdf]

Supplemental Online Content

Matson TE, Lapham GT, Bobb JF, et al. Validity of the Single-Item Screen–Cannabis (SIS-C) for cannabis use disorder screening in routine care. *JAMA Netw Open.* 2022;5(11):e2239772. doi:10.1001/jamanetworkopen.2022.39772

eFigure 1. Flow Diagram of Study Sample

eTable 1. Characteristics of the Eligible Primary Care Population (N=1688), Stratified by Severity of Past-Year Cannabis Use Disorder (CUD)

eAppendix. Performance of the SIS-C Across Sociodemographic Subgroups

eFigure 2. Receiver Operating Characteristic (ROC) Curves for the Single-Item Screen–Cannabis (SIS-C) Compared With the Reference Standard for Past-Year Cannabis Use Disorder (CUD), Stratified by Subgroups

eTable 2. Differences in Area Under the Receiver Operating Characteristic Curve (AUC) Estimates Comparing Performance of the SIS-C Between Demographic Subgroups and Performance SIS-C With Other Survey Measures

This supplemental material has been provided by the authors to give readers additional information about their work.

© 2022 Matson TE et al. *JAMA Network Open*.

eFigure1. Flow diagram of study sample

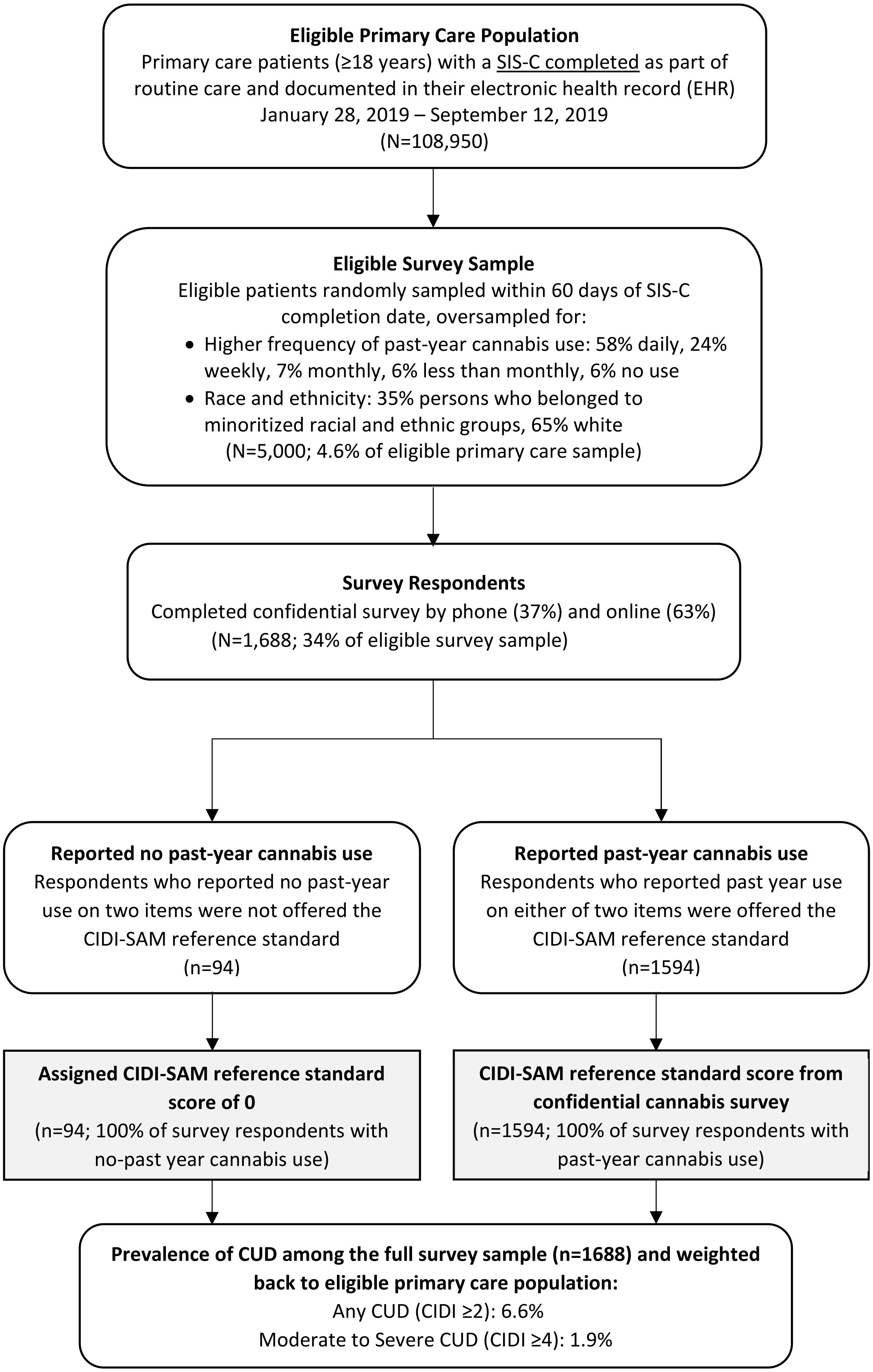

© 2022 Matson TE et al. *JAMA Network Open*.

eTable1. Characteristics of the eligible primary care population (N=1688), stratified by severity of past-year cannabis use disorder (CUD)

	No CUD (CIDI-SAM <2)[a]			Mild CUD (CIDI-SAM 2-3)			Moderate-Severe CUD (CIDI-SAM ≥4)		
	Unweighted No.	*Weighted %*	*(SE)*	*Unweighted No.*	*Weighted %*	*(SE)*	*Unweighted No.*	*Weighted %*	*(SE)*
**Age[b]**									
18-29	196	13.2	(32.9)	128	32.9	(7.1)	135	57.0	(4.3)
30-49	345	29.9	(52.6)	154	52.6	(8.9)	83	31.4	(3.9)
50-64	246	27.4	(4.0)	57	11.6	(2.8)	26	8.9	(1.9)
65+	283	29.5	(3.7)	25	2.8	(0.9)	10	2.7	(1.0)
**Sex or gender[b]**									
Female or women	562	56.6	(47.9)	192	47.9	(9.5)	107	40.7	(4.6)
Male or men	508	43.4	(52.1)	172	52.1	(9.5)	147	59.3	(4.6)
**Race[b]**									
American Indian or Alaska Native	31	0.4	(0.2)	5	0.4	(0.2)	4	1.3	(0.7)
Asian	65	10.0	(2.6)	21	5.8	(2.6)	13	7.2	(4.2)
Black	92	4.8	(1.8)	44	4.9	(1.2)	27	6.0	(1.2)
Native Hawaiian or Pacific Islander	24	1.7	(0.1)	5	16.9	(13.7)	7	1.7	(0.1)
White	777	75.0	(3.9)	247	64.5	(11.1)	168	65.2	(4.8)
Other/Unknown	81	8.2	(2.7)	42	7.5	(1.9)	35	18.7	(4.0)
**Hispanic Ethnicity[b]**	99	3.0	(6.6)	42	6.6	(2.4)	33	7.8	(1.6)
**Insurance[b]**									
Medicaid/Subsidized	125	5.6	(12.9)	54	12.9	(3.8)	31	8.6	(1.7)
Medicare	284	28.8	(3.1)	28	3.1	(0.9)	11	2.5	(0.8)
Commercial	613	63.7	(81.6)	264	81.6	(4.4)	195	81.4	(3.0)
Unknown	48	1.9	(2.4)	18	2.4	(0.8)	17	7.6	(2.5)
**Marital Status[c,d]**									
Married/Living with partner	649	64.2	(40.1)	193	40.1	(7.8)	124	50.0	(4.5)
Widowed	38	3.1	(0.6)	5	0.6	(0.3)	0	0.0	(0.0)
Divorced/Separated	117	9.5	(5.5)	32	5.5	(1.7)	17	5.2	(1.4)
Single/Never married	261	22.2	(53.7)	133	53.7	(8.7)	111	44.3	(4.5)
**Education[c,d]**									
≤ High school	182	12.1	(20.7)	84	20.7	(5.4)	53	23.5	(3.9)
Some college	421	38.3	(45.7)	145	45.7	(9.8)	99	34.4	(3.9)
≥ 4 years of college	460	48.7	(33.5)	134	33.5	(6.8)	100	41.7	(4.6)
**Employment[c,d]**									
Employed full time	584	54.1	(75.4)	239	75.4	(5.7)	165	67.0	(4.1)
Employed part time	92	13.1	(5.5)	37	5.5	(1.5)	23	6.8	(1.5)
Retired	256	23.3	(3.3)	25	3.3	(1.0)	17	4.3	(1.1)
Other	103	8.1	(8.9)	48	8.9	(3.2)	35	17.3	(3.8)
Unemployed	30	0.4	(6.7)	14	6.7	(3.3)	14	4.6	(1.3)
**EHR-Documented Past-Year Diagnoses[a]**									
Mental health diagnosis	340	25.8	(35.1)	156	35.1	(7.2)	116	39.4	(4.1)
SUD diagnosis	63	5.3	(3.4)	25	3.4	(1.0)	18	5.3	(1.3)

Abbreviations: CUD = cannabis use disorder; CIDI-SAM = Composite International Diagnostic Interview Substance Abuse Module; SUD = substance use disorder

[a] Participants who reported no past-year cannabis use on the survey were assigned a score of 0 on the CIDI-SAM.

[b] Data collected from electronic health records

[c] Data collected from confidential survey

[d] Subgroups do not sum to total N due to missing responses

© 2022 Matson TE et al. *JAMA Network Open*.

eAppendix. Performance of the SIS-C Across Sociodemographic Subgroups

For identification of any past-year CUD, performance of the SIS-C differed significantly across age (eFigure2). Compared to patients ≥50 years old (AUC 0.97), AUC was significantly lower for patients 30-49 (AUC 0.80; difference=0.17 [95% CI: 0.02-0.40]) and 18-29 (AUC 0.91; difference=0.06 [95% CI: 0.02-0.13]). There was no significant difference between patients 30-49 and 18-29. Performance of the SIS-C did not differ significantly by sex or gender or race and ethnicity (eTable2).

For identification of any moderate-severe CUD, performance of the SIS-C for identifying moderate-severe CUD differed significantly across age, race and ethnicity (eFigure2). Compared to patients ≥50 years old (AUC 0.98), AUC was significantly lower for patients 30-49 (AUC 0.95; difference=0.03 [95% CI: 0.01-0.5]) and 18-29 (AUC 0.91; difference=0.06 [95% CI: 0.03-0.13]). There was no significant difference between patients 30-49 and 18-29. Performance of the SIS-C was significantly lower for Hispanic patients (AUC 0.91) compared to non-Hispanic White patients (AUC 0.96; difference=0.05 [95% CI: 0.01-0.16]) but did not differ between non-Hispanic White and non-Hispanic Black patients (eTable2).

© 2022 Matson TE et al. *JAMA Network Open.*

eFigure2. Receiver Operating Characteristic (ROC) curves for the Single-Item Screen - Cannabis (SIS-C) compared to the reference standard for past-year cannabis use disorder (CUD), stratified by subgroups.

eFigure2 caption: When compared to any CUD (top row), the performance of the SIS-C differed significantly by age (a higher AUC for patients ≥50 compared to patients 30-49 and 18-29) but not by sex or gender or race and ethnicity. When compared to moderate-severe CUD (bottom row), the performance of the SIS-C differed significantly by age and race and ethnicity (a lower AUC for Hispanic patients compared to non-Hispanic White patients), but there was no significant difference by sex or gender.

© 2022 Matson TE et al. *JAMA Network Open*.

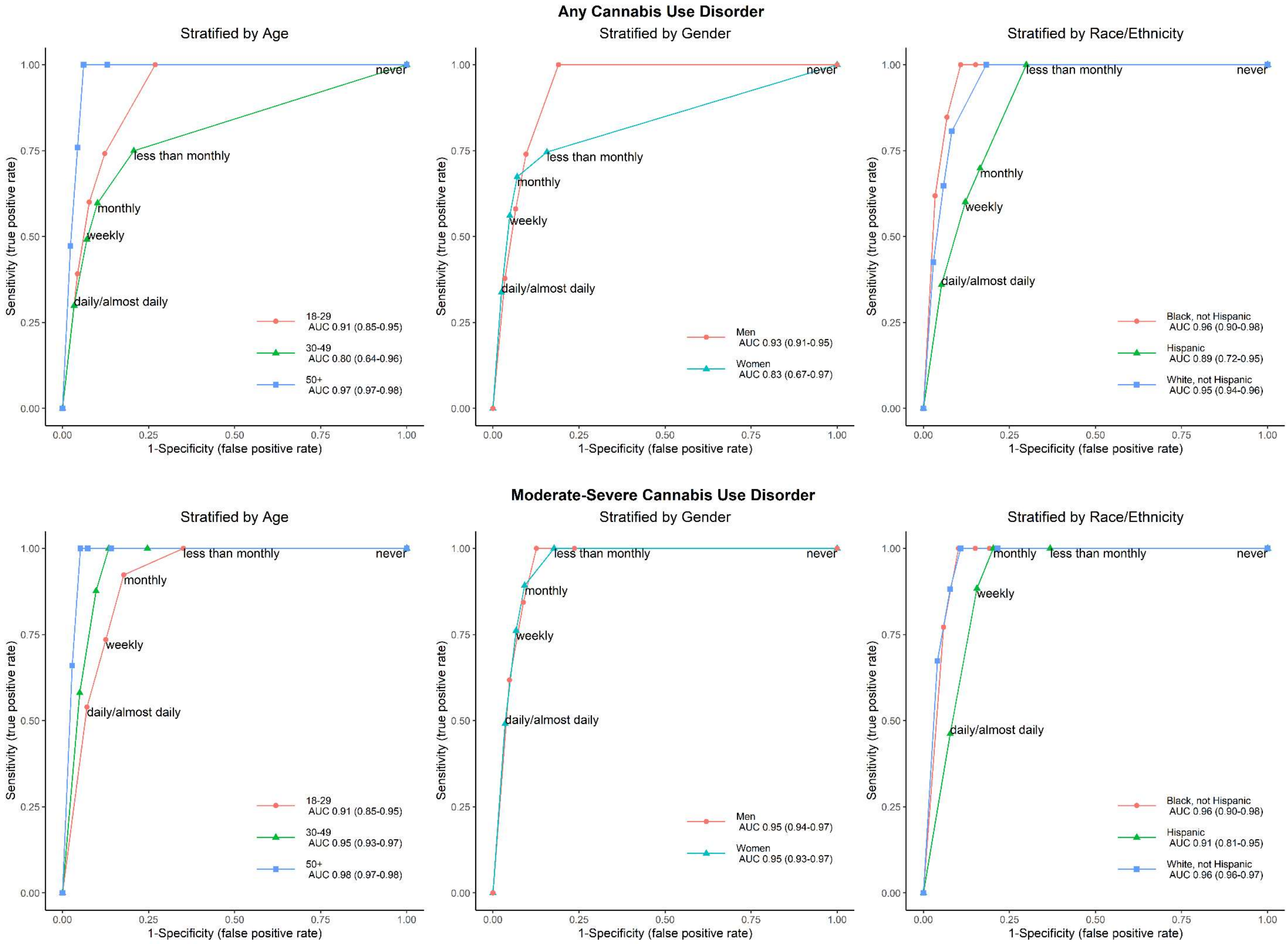

© 2022 Matson TE et al. *JAMA Network Open.*

eTable2. Differences in area under the receiver operating characteristic curve (AUC) estimates comparing: 1) performance of the SIS between demographic subgroups and 2) performance SIS to other survey measures

	**Any CUD[a]**		**Moderate-Severe CUD[b]**	
	Δ AUC[c]	*95% CI*[d]	*Δ AUC*[c]	*95% CI*[d]
Comparison of subgroups (reference vs. comparator)				
18-29 vs. 30-49	0.113	(-0.075, 0.276)	-0.037	(-0.105, 0.007)
18-29 vs. 50+	**-0.058**	**(-0.128, -0.02)**	**-0.064**	**(-0.130, -0.027)**
30-49 vs. 50+	**-0.171**	**(-0.330, -0.016)**	**-0.028**	**(-0.053, -0.008)**
Female or Women vs. Male or Men	-0.107	(-0.263, 0.050)	0.000	(-0.027, 0.026)
Black, not Hispanic vs. Hispanic	0.078	(-0.007, 0.245)	0.047	(-0.030, 0.155)
Black, not Hispanic vs. White, not Hispanic	0.017	(-0.039, 0.043)	0.004	(-0.022, 0.156)
Hispanic vs. White, not Hispanic	-0.062	(-0.007, 0.227)	**-0.051**	**(0.010, 0.156)**

Abbreviations: CUD = cannabis use disorder; Δ = difference; AUC = area under the receiver operating characteristic curve; CI = confidence interval
[a] Endorsed ≥2 criteria on the Composite International Diagnostic Interview (CIDI)
[b] Endorsed ≥4 criteria on the Composite International Diagnostic Interview (CIDI)
[c] A positive difference indicates that the reference group or measure had better performance than the comparator; a negative difference indicates that the comparator group had better performance than the reference group.
[d] 95% CI for the difference in AUC estimates obtained using nonparametric bootstrapping. 95% CIs that do not contain zero indicate a significant between-group difference and denoted with **bold** font.

© 2022 Matson TE et al. J*AMA Network Open*.
